# Effect of acute exercise on patella tendon protein synthesis and gene expression

**DOI:** 10.1186/2193-1801-2-109

**Published:** 2013-03-13

**Authors:** Kasper Dideriksen, Ann Kathrine Ryberg Sindby, Michael Krogsgaard, Peter Schjerling, Lars Holm, Henning Langberg

**Affiliations:** 1 Institute of Sports Medicine, Department of Orthopedic Surgery M81, Bispebjerg Hospital, Bispebjerg Bakke 23, DK-2400 Copenhagen, NV, Denmark; 2 Center for Healthy Aging, Faculty of Health and Medical Sciences, University of Copenhagen, Blegdamsvej 3, DK-2200 Copenhagen, N, Denmark; 3 Institute of Sports Traumatology, Department of Orthopedic Surgery M51, Bispebjerg Hospital, Bispebjerg Bakke 23, DK-2400 Copenhagen, NV, Denmark

**Keywords:** Tendon Collagen FSR, TGF-β-1, CTGF, Type I collagen, Type III collagen

## Abstract

Evidence suggests that habitual loading can result in patellar tendon hypertrophy, especially at the proximal and distal parts of the patellar tendon. The underlying protein kinetic changes and its regulation remains controversial and human data, investigating this topic, are limited. The present study investigated how acute exercise affects growth factor production and collagen fractional synthetic rate in patellar tendon tissue from patients undergoing an anterior cruciate ligament reconstruction operation. The operation was performed by use of the bone-patellar tendon-bone method under spinal anesthesia.

Twelve subjects were randomized to one of two groups: a control group or an exercise group (1-hr unilateral knee-extension 67% of W_max_ 24 hours before operation). Two hours before the anterior cruciate ligament operation a flooding-dose of L-[1-^13^C]proline was given. Tissue from the most proximal part of the patellar tendon was obtained during the operation. Tendon collagen fractional synthetic rate and mRNA concentrations of TGF-β-1, CTGF, and type I and III collagen were measured.

CTGF and type I collagen expression were higher in the exercise group compared to the control group (p < 0.05). Type III collagen expression (p = 0.11), TGF-β-1 expression (p = 0.34), and collagen fractional synthetic rate (p = 0.26) did not differ between groups.

Although the expression of CTGF and type I collagen were higher, the patellar tendon collagen fractional synthetic rate was not correspondingly higher after exercise. The elevated CTGF expression in the exercise group indicates that the TGF-beta pathway could be an important link between mechanical loading and stimulation of tendon tissue type I collagen expression.

## Background

The tendons transmit force from muscles to bones and are, thus, an essential part in movement. Human tendons consist mainly of fibrillar type I and type III collagen proteins, with type I collagen as the predominant part (Kjaer, 2004). The collagen molecules are produced in tendon fibroblasts, which interact with the extracellular matrix to form the connective tissue network of tendons (Kjaer et al., 2009). Mechanical stress/strain can be applied to the tendons through its connection with the extracellular matrix and cytoskeleton of the muscle tissue and the tendon tissue is able to adapt both in a structural and functional ways to external stimuli (Kjaer, 2004). In accordance, habitual physical training has been shown to induce hypertrophy and improved stiffness of human tendons (Kongsgaard et al., 2005; Kongsgaard et al., 2007; Couppé et al., 2008; Rønnestad et al., 2012).

Even though high-intensity or high-volume physical exercise can stimulate tendon collagen protein turnover, limited information exists regarding the underlying mechanisms that mediate this exercise-induced response. On the acute basis, the growth factor and collagen mRNA gene expression has previously been shown to increase following exercise in both humans and rats (Langberg et al., 1999; Heinemeier et al., 2003; Heinemeier et al., 2007; Olesen et al., 2006). Transforming growth factor-β-1 (TGF-β-1) and its downstream mediator in fibroblastic cells, connective tissue growth factor (CTGF), may be a part of the link between mechanical loading and tendon tissue collagen protein turnover (Frazier et al., 1996; Grotendorst, 1997; Duncan et al., 1999; Moussad & Brigstock, 2000; Hishikawa et al., 2001; Schild & Trueb, 2002; Chiquet et al., 2003; Nakama et al., 2006; Chen et al., 2008). However, it is not a universal finding that the tendon gene expression does increase after exercise. Recently, it was reported that the human patellar tendon gene expression of TGF-β-1, CTGF, type I collagen, and type III collagen was unaffected 26 hrs following one hr of strenuous kicking exercise (Heinemeier et al., 2011). Additionally, type I and III collagen mRNA was not up regulated 24 hrs after resistance exercise (Sullivan et al., 2009). But considering the sparse information that exists regarding human tendon gene expression in response to exercise, it seems important to further investigate the effect of exercise on human tendon tissue.

The human tendon collagen protein turnover has been shown to be stimulated following exercise with measurements of the aminoterminal and carboxyterminal propeptide of procollagen type I (PINP and PICP) concentrations in human peritendinous tissue and the stable isotope collagen incorporation method (Langberg et al., 1999; Langberg et al., 2001; Heinemeier et al., 2003; Miller et al., 2005; Miller et al., 2007; Hansen et al., 2008). Especially, the patellar tendon collagen fractional synthetic rate (FSR) has previously been found increased from 6 to 72 hrs following one hr of strenuous kicking exercise (Miller et al., 2005). On the other hand, not all studies have reported an increased tendon collagen FSR following this type of exercise (Hansen et al., 2009b; Petersen et al., 2011).

Patellar tendon hypertrophy observed with habitual physical training does primarily take place at the proximal and distal portions of the tendon (Kongsgaard et al., 2007; Couppé et al., 2008). Furthermore, the human studies which do not report any change in gene expression or collagen FSR have all analysed tendon tissue from the middle or a more proximal portion of the patellar tendon (Hansen et al., 2009b; Sullivan et al., 2009; Heinemeier et al., 2011; Petersen et al., 2011). Thus, we took the approach to measure the effect of acute exercise on anterior cruciate ligament (ACL) patients undergoing an ACL reconstruction operation. With this approach, human patellar tendon tissue could be obtained from the most proximal part the tendon. The human patellar tendon gene expression alongside tendon collagen FSR were measured 24-hours after a 1-hr strenuous kicking exercise bout, as this should be the time point where the tendon collagen protein synthesis peaks (Miller et al., 2005). We hypothesized that the expression of growth factors and collagen, as well as the synthesis of patellar tendon collagen proteins would be higher following exercise compared to under resting conditions.

## Methods

*Subjects* 6 women and 6 men (age 31 ± 2 yrs), who had a reconstruction of the anterior cruciate ligament (ACL) planned, were included in the study (Table 1). In all subjects, the ACL rupture did occur at least three months prior to the ACL reconstruction operation. The subjects had a moderate activity level before the ACL rupture, and some subjects did reduce their activity levels as a result of the injury. None of the subjects did participate in any supervised pre-operative therapy. No signs of knee joint effusion, arthrofibrosis or patellar tendon tendinopathy were observed in any of the included subjects. Additionally, none of the subjects displayed meniscus or collateral ligament tears.

**Table 1:** **Subject characteristics**

Experimental group	Control	Exercise	***P*** Value
Variable			
Sex distribution (M/F)	3/3	3/3	-
Age (yrs)	31 ± 3	32 ± 3	NS
Height (m)	1.73 ± 0.04	1.75 ± 0.05	NS
Weight (kg)	75.3 ± 4.8	73.5 ± 4.2	NS
BMl (kg/m^2^)	25.1 ± 0.9	23.9 ± 0.4	NS
67% of max workload (watt)	-	44 ± 8	-

Data are mean ± SEM. An unpaired and two-tailed t-tests was conducted to determine group differences. NS: not significant; Control: no exercise; Exercise: 1-hr unilateral kicking at 67% of maximal workload 24 hours before trial.

Subjects enrolled in the study underwent an examination, which included an evaluation of the history of medical intake and diseases, physical activity level, and dietary/smoking habits. Subjects were excluded if they had a BMI below 20 or above 27.5 kg/m^2^, suffered from cardiovascular or metabolic diseases, or took any kind of medication that could influence the outcome of the study. The female subjects were all menstruating regularly. Due to the study coordination process with limited flexibility in the operational schedule we choose not to control for the menstrual cycle during the study. Keeping an even distribution of sex, age, and body composition in each group, the subjects were randomly allocated (by envelopes) to one of two groups: A non-exercise group (control) and a group exercising 24 hrs prior to the operation (exercise). All subjects were carefully informed in accordance with the Declaration of Helsinki II before they gave their written consent to participate in the study. The local ethics committee of Copenhagen and Frederiksberg approved the study (KF 11-053/04).

### Pretest and food intake

At least two weeks before carrying out the study, subjects had their maximum workload (W_max_) determined on a one-legged modified Krogh cycle ergometer, as described in detail elsewhere (Miller et al., 2005; Hansen et al., 2008).

During the three days before the operation the subjects were instructed to follow their normal eating pattern and refrain from physical activity besides normal daily living-activities. Besides the completion of the exercise intervention, none of the included subjects did perform any physical activity during the three days prior to the operation.

### Experimental protocol

**Figure 1:**
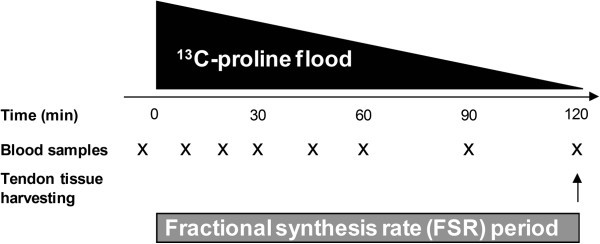
**Experimental protocol for the acute studies.** 750 mg ^13^C-proline and 3250 mg ^12^C-proline was injected over 3 min. Venous blood samples were drawn at 10- to 30-min intervals after the bolus injection. The tendon fractional synthetic rate (FSR) was measured over a 2 hr-period. Patellar tendon tissue was harvested during the operation. In the exercise group, a 1-hr unilateral exercise bout was completed 24 hours before tendon tissue was harvested.

As illustrated in Figure 1, the overall design of the acute studies was identical. However, only the exercise group performed a 1-hr unilateral kicking exercise bout at a workload of 67% of their W_max_ and frequency of 35 kicks · min^-1^ with the injured leg, resulting in 2100 concentric contractions in total. The exercise bout was completed 24 hrs before the patellar tendon graft was removed. All the subjects in the exercise group completed the exercise bout with the prescribed intensity.

The acute studies were started between 7 am and 9 am on the day of the ACL reconstruction operation and all subjects were fasting overnight. Two catheters were inserted into veins on opposite forearms for isotope infusion and blood sampling from which a blood sample was drawn for measurement of background isotope enrichment. Two hours before operation, a flooding dose of L-[1-^13^C]proline (Cambridge Isotopes Laboratories, Andover, MA; flooding dose = 750 mg ^13^C-proline and 3250 mg ^12^C-proline) was administered over three min. As illustrated in Figure 1, blood samples were drawn at 10- to 30-min intervals after the bolus injection for determination of the ^13^C-proline enrichment in plasma, measured as the tracer/trace ratio.

**Figure 2:**
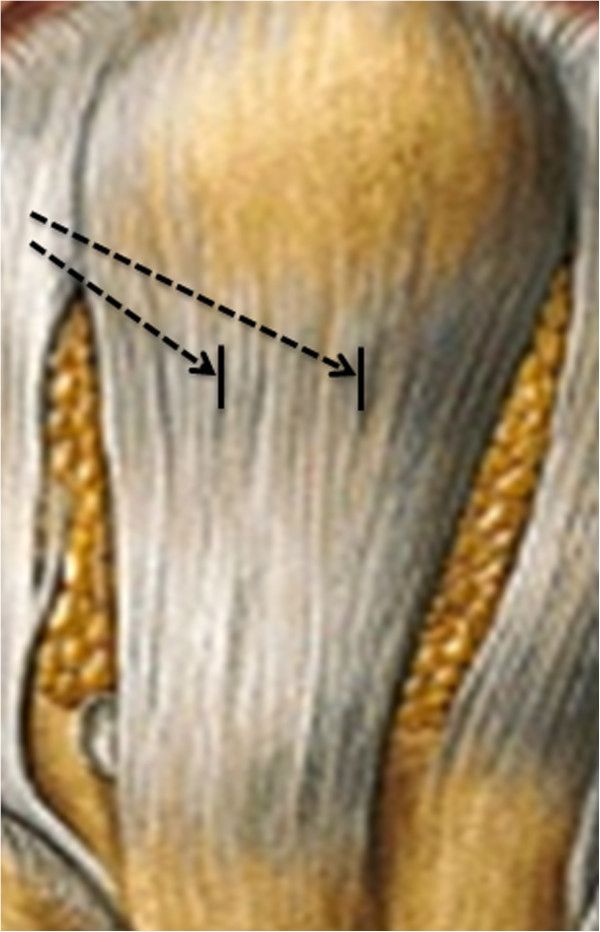
**Depiction of the patellar tendon.** The patellar tendon tissue obtained during the operations was located at the most proximal portion of the tendon, right next to the tendon insertion as illustrated by the black arrows.

The bone-patellar tendon-bone method was used for the ACL reconstruction (Meisterling et al., 1993). With this method, the mid third of the patellar tendon and a small piece of the connected patella and tibia bones (the graft) was harvested under spinal anesthesia. Arthroscopic autograft reconstruction of the ACL is one of the most abundant surgical procedures in sports medicine, especially in young athletes (Xergia et al., 2011). Normally, the blood circulation to the knee is stopped by a tourniquet during the operation, but to sustain the physiological conditions and to ensure the delivery of the stabile isotope ^13^C-proline to the patellar tendon as long as possible (until the removal of the graft) a tourniquet was not used during the operation. All operations were performed by the same surgeon, who was blinded to which group the subjects were randomized to. Before inserted into the knee the graft was processed, leaving sufficient amount of patellar tendon tissue for measurement of collagen protein FSR and gene expression of TGF-β-1, CTGF, type I collagen, and type III collagen. Importantly, the patellar tendon tissue obtained during the operations was located at the most proximal portion of the tendon, right next to the tendon insertion (Figure 2).

### Stable isotope analyses

To determine the plasma ^13^C-proline enrichment, the blood samples were prepared as previously described and analyzed by gas-chromatography mass-spectrometry (GC: Trace GC 2000 series, MS: Automass Multi, Thermo Quest Finnigan, Paris, France) (Hansen et al., 2009b). Plasma ^13^C-proline enrichment was used as an acceptable alternative of the true intracellular precursor pool, prolyl-tRNA, since the flooding technique is assumed to equilibrate all free amino acid pools, making the plasma and the tissue-free proline labelling indistinguishable.

To determine ^13^C-proline enrichment in tendon collagen protein, we used a previously described procedure (Hansen et al., 2009b). All the protein fractions were hydrolyzed in 6 M HCl at 110°C for 18 hrs, and the amino acids were purified by acidic cation exchange resin columns (Dowex AG-50W, Bio-Rad, Copenhagen, Denmark). The samples were then n-acetyl n-propyl (NAP) derivatized and analyzed on the gas-chromatography combustion isotope ratio mass-spectrometry analyzer (DeltaPlus XL, Thermo Finnigan, Bremen, Germany) (Hansen et al., 2009b).

### Calculations

**Figure 3:**
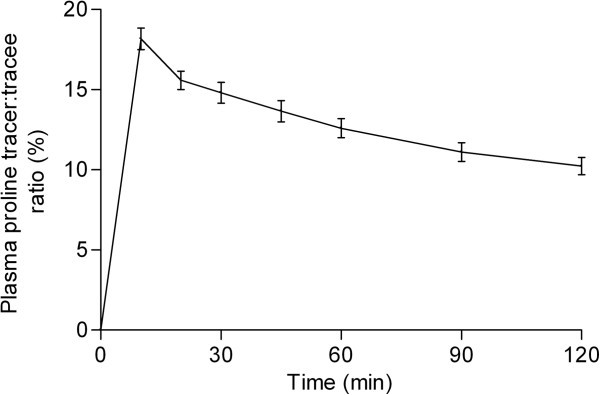
**Mean (± SEM) venous plasma proline tracer:tracee ratio (%) enrichments throughout the fractional synthetic rate measuring period.** The individual values of the area under the plasma proline enrichment curves were used for calculation of tendon collagen FSR.

The collagen FSR were calculated from the ^13^C-proline incorporation into tendon protein using the standard precursor-product method: FSR (% · h^-1^) = ΔE_product_ · (E_precursor_ · Δtime)^-1^ · 100%, where ΔE_product_ represents the change in protein-bound tracer enrichment from the calculated background value to the tendon tissue sample. As previously described (Doessing et al., 2010), the background plasma protein ^13^C-proline enrichment from each individual was used to establish the natural abundance of ^13^C-proline in collagen tissue. Δtime is the time period of tracer incorporation, and E_precursor_ is the area under the individual plasma proline enrichment curves during the time period of tracer incorporation (Figure 3).

### RNA extraction

Total tendon RNA was extracted according to the previously described method for tendon tissue (Heinemeier et al., 2007).

### Real time PCR

**Figure 4:**
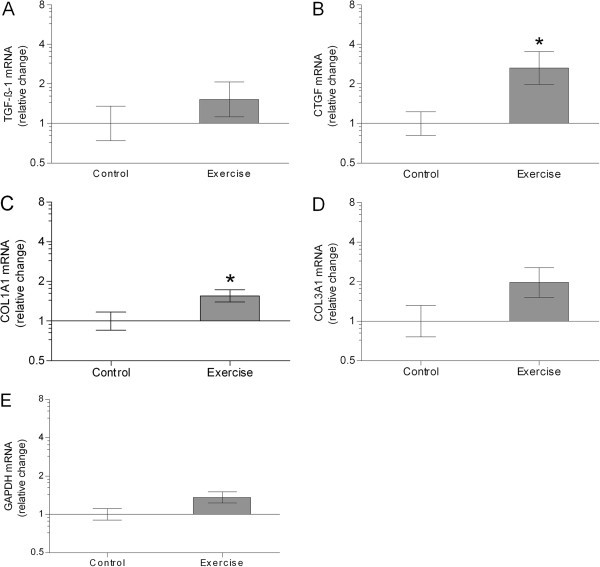
**Geometric mean (± back-transformed SEM) of TGF-β-1 (A), CTGF (B), COL1A1 (C), COL3A1 (D), GAPDH (E) mRNA, normalized to RPLP0 mRNA, in Control (no bars) and Exercise (grey bars).** All data are presented as fold changes between the two groups. That is the changes relative to the mean of all control values. Data were analyzed with unpaired and two-tailed t-tests. For TGF-β-1 (p = 0.34), no difference between groups was observed. Significant higher CTGF and COL1A1 mRNA levels were observed in the exercise group compared to the control group (p < 0.05). For COL3A1 (p = 0.11), the difference between groups was not significant. Control: no exercise; Exercise: 1-hr unilateral kicking at 67% of maximal workload 24 hours before trial.

100 ng of total tendon RNA was converted to cDNA and measured by real time PCR as described previously, using the same primers (Mackey et al., 2011). GAPDH mRNA was normalized with RPLP0 mRNA to validate RPLP0 mRNA as an internal reference for all mRNA levels. However the GAPDH-RPLP0 ratio (Figure 4E) was somewhat higher, although not significant (p = 0.08), in the exercise group compared to the control group. If the difference is real, we consider it more likely that, the difference between GAPDH mRNA and RPLP0 mRNA reflects an increased GAPDH mRNA expression, rather than a decreased RPLP0 mRNA expression, following the strenuous exercise bout. We therefore conclude that, the most reasonable was to normalize all other targets with RPLP0.

### Statistical analysis

All values are presented as means ± standard error of the mean (SEM), except the mRNA levels which are log-transformed for statistical analysis and therefore given as geometric mean ± back-transformed SEM. Statistical analysis for all comparisons of subject characteristics between the two groups were performed using parametric, unpaired, and two-tailed t-tests. Analyses of tendon FSR and log-transformed mRNA data between groups were carried out using parametric, unpaired, and two-tailed t-tests. Level of significance was set at p < 0.05, and all analyses were done using Prism 4.0 (GraphPad Software Inc., San Diego, California).

## Results

### Subject characteristics

Subject characteristics are presented in Table 1. There were no differences in any of the variables between the two groups. All subjects tolerated the strenuous exercise bout.

### TGF-β-1, CTGF, COL1A1, COL3A1, and GAPDH mRNA

Patellar tendon tissue mRNA levels of TGF-β-1, CTGF, COL1A1, COL3A1, and GAPDH are shown in Figure 4. All data are presented as fold changes relative to the mean of the control values. The level of TGF-β-1 did not differ between groups (p = 0.34). In the exercise group the CTGF and COL1A1 mRNA levels were higher compared to in the control group (p < 0.05). The level of COL3A1 did not differ significantly between groups (p = 0.11).

### Tendon collagen protein synthesis

**Figure 5:**
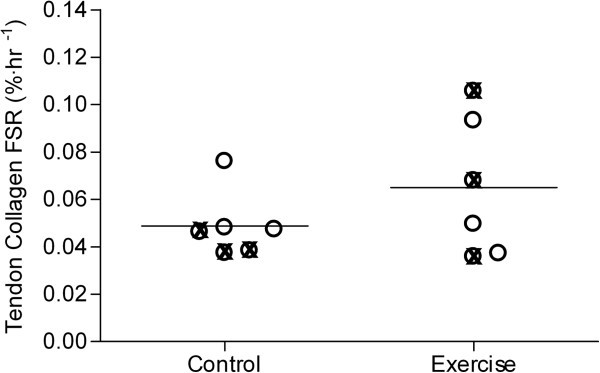
**Distribution of tendon collagen protein fractional synthetic rate (FSR; %·hr**^**-1**^**) in the two groups.** To illustrate female and male values, different symbols are used, **⊗** = females and **O** = males. An unpaired and two-tailed t-tests was conducted to determine the difference between groups. The difference between groups was not significant (p = 0.26). Control: no exercise; Exercise: 1-hr unilateral kicking at 67% of maximal workload 24 hours before trial.

The patellar tendon collagen FSR with venous ^13^C-proline enrichment as the precursor (Figure 3) is presented in Figure 5. The collagen FSR was (0.049 ± 0.006% · hr^-1^) in the control and (0.065 ± 0.012% · hr^-1^) in the exercise showing no significant difference between groups (p = 0.26).

## Discussion

The most important findings in the present study was, that the level of CTGF and collagen type I mRNA expression was higher 24 hrs after exercise, which though, was not translated into a higher collagen protein synthesis rate. Longitudinal studies have reported that habitual loading primarily stimulates patellar tendon growth at the proximal and distal portions of the tendon, right next to the tendon insertions (Kongsgaard et al., 2007; Couppé et al., 2008). With the present study design the tendon tissue could be obtained from a more proximal portion of the patellar tendon compared to the few previous acute human studies (Sullivan et al., 2009, Heinemeier et al., 2011) that has adressed this topic. This might explain the observed effect of exercise on human patellar tendon gene expression.

The finding of a higher collagen I expression in the exercise group compared to the control group indicates that the tendon genes which codes for structural proteins does increase following exercise. This is supported by studies done in rat tendons, showing that both collagen I and collagen III expression does increase with different types of mechanical loading (Olesen et al., 2006; Heinemeier et al., 2007). In humans, not much research has been published regarding the acute effect of exercise on tendon collagen gene expression. One study reported, though, that the collagen I and II expression did not change 24 hrs after resistance exercise in young men and women (Sullivan et al., 2009). However, the exercise protocol used by Sullivan et al. ( 2009) differs from our exercise protocol in exercise mode, duration, and intensity, which may explain the lack of difference in collagen gene expression following exercise (Sullivan et al., 2009). More recently, a study performed on young men completing a similar exercise protocol as in the present study, did not find any effect of exercise on patellar tendon growth factor or collagen expression 26 hrs after exercise (Heinemeier et al., 2011). Since the exercise protocol and the analytic methods were similar in the two studies, the different findings cannot be explained by such factors. Furthermore, it is unlikely, that the 2-hr longer period from exercise to tendon tissue sampling in Heinemeier et al. ( 2011) can explain the difference compared to the present study. The tendon tissue was obtained with the automatic tendon biopsy method (the Bard Monopty Biopsy Instrument) from a proximal portion of the patellar tendon in Heinemeier et al. ( 2011). In the present study, the tendon tissue was sampled from a more proximal portion of the tendon, right next to the tendon insertion. It has been reported that the patellar tendon growth primarily takes place at the proximal and distal portions of the tendon with habitual loading (Kongsgaard et al., 2007; Couppé et al., 2008). As proposed by Kongsgaard et al. ( 2007), the compressive load in these insertional regions possibly stimulates the synthesis of new extracellular matrix proteins. Based on these longitudinal findings, it could be speculated that the tendon transcription may primarily take place in the proximal and distal portion of the patellar tendon. Hence, the slightly different methods and locations of tendon tissue sampling applied in Heinemeier et al. ( 2011) compared to in the present study could have induced the contrasting results regarding the collagen type I expression.

Due to the limited number of subjects and the unpaired study design, the statistical power was rather low in the present study. Thus, we cannot exclude that the change in collagen I expression in the present study was due to a type I error. Furthermore, as it was very difficult to recruit the ACL patients and complete the operational-scheduled studies, both men and women were included in the study. Even though the two groups were equal with regard to age, body composition, and sex-distribution, the inclusion of both men and women could potentially have obscured the study outcome. It has been shown that the patellar tendon mRNA level of collagen III is significantly higher in women compared to men at rest (Sullivan et al., 2009). Additionally, patellar tendon collagen FSR has been reported to be lower in young women compared to age-matched men both at rest and following exercise (Miller et al., 2007). However, increased concentrations of the indirect marker for tendon collagen protein synthesis, PINP, have previously been measured in local patellar tendon tissue dialysate in young women 24 hrs (Hansen et al., 2008) and 72 hrs (Miller et al., 2007) after acute kicking exercise, which indicates that the female patellar tendon is able to respond to acute exercise. Nevertheless, we decided to pool all the individual FSR and gene expression values according to gender category, which should reveal any possible gender-differences in these parameters. No indications of gender-differences in collagen FSR (p = 0.82) and target gene expressions (the lowest value: p = 0.62) were found with these calculations. In accordance, no indications of a gender-effect could be observed from the individual tendon collagen FSR values displayed in Figure 5.

Based on previous findings (Miller et al., 2005) we did hypothesize to find a higher collagen FSR in the exercise group, as the exercise protocols used in Miller et al. ( 2005) and the present study are comparable. Furthermore, we choose the 24 hrs post exercise time point for tendon tissue sampling, as the patellar tendon FSR was found to increase the most at that time point (Miller et al., 2005). However, no difference was found on tendon collagen FSR in the present study. The mixture of men and women within groups did not seem to explain the contrasting collagen FSR results compared to those reported in Miller et al. ( 2005). Instead, a fairly large inter-subject variation was observed on tendon collagen FSR (Figure 5), with only 2 out of 6 subjects responding to the exercise bout. Since we cannot exclude that a type II error did occur, we performed a post-study power calculation with our measured inter-subject variation and difference between means, which indicated that 26 subjects should have been included in each group for the collagen FSR to increase significant with exercise. Hence, the effect of the exercise seems to be less consistent than hypothesized. In accordance with the present observations, the tendon collagen FSR was recently shown not to change 24 hrs after a similar kicking exercise bout in elderly men and women with osteoarthritis (Petersen et al., 2011). As discussed by Heinemeier et al. ( 2011), it is possible that the stimulating effect of the strenuous kicking exercise bout on patellar tendon FSR may be overestimated as a result of the study design in Miller et al. ( 2005) (see (Heinemeier et al., 2011) for more details).

It has previously been shown that changes in PINP does not reflect the simultaneously incorporation of tendon collagen protein into the final tendon structure in both men and women (Miller et al., 2005; Miller et al., 2007; Hansen et al., 2009a). Additionally, it has been proposed that some of the newly synthesized collagen molecules enter the pool of free collagen from where they may be catabolized or used for tendon protein synthesis at a later time point (Kjaer, 2004). This may explain the present findings of a higher tendon collagen type I mRNA expression without a correspondingly higher tendon collagen FSR.

Results obtained *in vitro* suggest that CTGF acts as a downstream mediator of TGF-β-1 in fibroblastic cells (Frazier et al., 1996; Grotendorst, 1997; Duncan et al., 1999; Moussad & Brigstock, 2000; Chiquet et al., 2003). Hence, the present observations of a higher CTGF expression after exercise may suggest that the TGF-β-1 expression has been increased at a time point earlier than 24 hrs after exercise. *In vivo* results indicate that TGF-β-1 is up regulated following exercise (Heinemeier et al., 2003; Heinemeier et al., 2007). However, Heinemeier et al. ( 2007) measured TGF-β-1 expression in rat tendons following resistance exercise, whereas Heinemeier et al. ( 2003) measured TGF-β-1 protein concentration in human plasma and Achilles tendon dialysate after uphill running exercise. The running exercise contains eccentric muscle contractions and may have resulted in different tendon stress/strain compared to concentric contractions performed in the present study. Hence, it is difficult to directly compare these results with the present results.

In different cell types, CTGF has been shown to be regulated by mechanical stress and involved in conversion of mechanical stimuli into biochemical effects, by increasing the collagen I expression and the synthesis of extracellular matrix proteins (Hishikawa et al., 2001; Schild & Trueb, 2002). On the other hand, increased collagen I and unchanged CTGF expressions have been reported after resistance exercise in rat Achiles tendons (Heinemeier et al., 2007). However, the fairly low-volume loading protocol used by Heinemeier et al. ( 2007) did possibly not induce enough stress/strain to stimulate the CTGF expression. In support of this, 80 hrs of repetitive loading lead to increased CTGF production (an increased amount of CTGF positive cells) in rabbit tendons (Nakama et al., 2006). Moreover, this strenuous loading model did induce tendon microtear formation (Nakama et al., 2005), which may have stimulated the expression of CTGF as this growth factor has been shown to be involved in tendon healing processes (Moussad & Brigstock, 2000; Chen et al., 2008). Since it was not examined, it cannot be excluded that tendon microtear formation could be responsible for the increased CTGF expression observed in the present study. However, the present finding of an unchanged collagen III expression indicates that the exercise bout did not damage the tendon seriously, since collagen III expression has been shown to be up regulated in injured tendon tissue (Eriksen et al., 2002; Lui et al., 2010). It could be speculated, though, that the unchanged collagen III expression may be due to a type II error, since it almost tended to be higher in Exercise compared to Control (p = 0.11). A post-study power calculation, using the measured inter-subject variation and mean difference, indicated that 13 subjects should have been included in each group for the collagen III expression to be significantly higher in Exercise compared to Control.

## Conclusions

In summary, patellar tendon mRNA levels of CTGF and collagen I were increased 24 hours after one hr of strenuous kicking exercise. The higher collagen I expression was not translated into a higher tendon collagen protein FSR though. The elevated CTGF expression in the exercise group indicates that the TGF-beta pathway could be an important link between mechanical loading and stimulation of tendon tissue type I collagen expression. Previous studies have reported that habitual loading primarily stimulates patellar tendon growth at the proximal and distal portions of the tendon, right next to the tendon insertions. So far, very few studies have investigated the effect of acute exercise on human patellar tendon gene expression. With the present study design, patellar tendon tissue could be obtained from a more proximal portion of the tendon compared to what has been done in the few previous human studies. This might explain the exercise-induced effect on human patellar tendon gene expression, which contrasts previous findings. On the other hand, since the statistical power of the present study was rather low, it cannot be excluded that the exercise-induced effect was caused by a type I error. Hence, future studies should address the exercise adaptive response of the human patellar tendon at different locations of the tendon.

## Authors’ information

Henning Langberg is now at CopenRehab, Section of Social Medicine, Department of Public Health, Faculty of Heath Sciences, University of Copenhagen, Copenhagen, Denmark.

## Abbreviations

ACL: Anterior cruciate ligament
CTGF: Connective tissue growth factor
FSR: Fractional synthetic rate
PICP: Carboxyterminal propeptide of type I collagen
PINP: Aminoterminal propeptide of type I collagen
TGF-β-1: Transforming growth factor-β-1
W_max_: Maximum workload.
